# Early Metastatic Breast Cancer in a Nonverbal Autistic Patient; A Complex Presentation

**DOI:** 10.51894/001c.144205

**Published:** 2025-09-30

**Authors:** Mariam Ahsan

**Affiliations:** 1 Internal Medicine Detroit Medical Center-Sinai Grace Hospital

## 58

### INTRODUCTION

Breast cancer in younger individuals is uncommon but clinically significant, especially in cases involving genetic predispositions like BRCA1/2 mutations. Diagnosing malignancies in nonverbal, neurodivergent patients presents challenges due to communication barriers and atypical symptom presentations. This report describes a case of metastatic breast cancer in a 38-year-old nonverbal autistic female, initially presenting with severe abdominal pain. The case highlights the importance of a multidisciplinary approach and genetic evaluation in guiding treatment.

### CASE DESCRIPTION

A 38-year-old nonverbal autistic female was admitted with progressive left lower quadrant abdominal pain, weakness, and distress. Symptoms followed treatment for a urinary tract infection. The patient localized the pain by touching the affected area, with moderate tenderness, impaired ambulation, and recent weight loss.

On examination, she was hemodynamically stable. Abdominal evaluation revealed left lower quadrant tenderness. Further assessment identified a firm, nonmobile ~4 cm mass in the left breast (12–3 o’clock position) with left axillary lymphadenopathy. A ~2 cm right breast mass (3 o’clock position) was also detected. Labs showed hypercalcemia (11.2 mg/dL) and anemia (hemoglobin 10.7 g/dL).

Imaging revealed extensive metastases. CTAP demonstrated mixed lytic and blastic bone lesions, including a 7.8 cm sacral mass causing spinal canal stenosis. CT chest showed diffuse lytic spinal lesions, a mild T11 compression fracture, and bilateral axillary adenopathy. MRI confirmed spinal and sacral metastases. Biopsy of the right iliac crest showed metastatic adenocarcinoma of breast origin. Immunohistochemistry confirmed ER-positive, PR-negative, HER2-negative disease.

Management included a multidisciplinary team. Tamoxifen (20 mg daily) was initiated, with plans for CDK 4/6 inhibitors and luteinizing hormone-releasing hormone (LHRH) agonists. Pain was controlled with Norco, Naproxen, and Gabapentin, while hypercalcemia was managed with intravenous hydration. Genetic counseling was arranged to assess hereditary cancer syndromes.

### DISCUSSION/CONCLUSION

Diagnosing malignancy in nonverbal patients with neurodevelopmental disorders is challenging due to atypical presentations and diagnostic delays. Abdominal pain initially masked metastatic breast cancer, emphasizing the need for thorough evaluation. Multidisciplinary care was essential for oncologic treatment, pain management, and genetic counseling. This case highlights the necessity of considering malignancy in neurodivergent patients with nonspecific symptoms and reinforces structured diagnostic and management approaches to improve outcomes.

